# Public Beliefs about Antibiotics, Infection and Resistance: A Qualitative Study

**DOI:** 10.3390/antibiotics2040465

**Published:** 2013-11-05

**Authors:** Pauline Norris, Kerry Chamberlain, Kevin Dew, Jonathan Gabe, Darrin Hodgetts, Helen Madden

**Affiliations:** 1 School of Pharmacy, University of Otago, Box 56, Dunedin 9054, New Zealand; 2 School of Psychology, Massey University Albany, Private Bag 102904, North Shore, North Shore City 0745, New Zealand; E-Mails: k.chamberlain@massey.ac.nz (K.C.); hmadden2012@gmail.com (H.M.); 3 School of Social and Cultural Studies, Victoria University of Wellington, Box 600, Wellington 6140, New Zealand; E-Mail: kevin.dew@vuw.ac.nz; 4 Centre for Criminology and Sociology, Royal Holloway, University of London, Egham, Surrey TW20 0EX, UK; E-Mail: J.Gabe@rhul.ac.uk; 5 School of Psychology, University of Waikato, Private Bag 3105, Hamilton 3240, New Zealand; E-Mail: dhodgetts@waikato.ac.nz

**Keywords:** public knowledge, lay attitudes, qualitative, antibiotics

## Abstract

We aimed to gain an in-depth understanding of public views and ways of talking about antibiotics. Four focus groups were held with members of the public. In addition, 39 households were recruited and interviews, diaries of medicine taking, diaries of any contact with medication were used to explore understanding and use of medication. Discussions related to antibiotics were identified and analyzed. Participants in this study were worried about adverse effects of antibiotics, particularly for recurrent infections. Some were concerned that antibiotics upset the body’s “balance”, and many used strategies to try to prevent and treat infections without antibiotics. They rarely used military metaphors about infection (e.g., describing bacteria as invading armies) but instead spoke of clearing infections. They had little understanding of the concept of antibiotic resistance but they thought that over-using antibiotics was unwise because it would reduce their future effectiveness. Previous studies tend to focus on problems such as lack of knowledge, or belief in the curative powers of antibiotics for viral illness, and neglect the concerns that people have about antibiotics, and the fact that many people try to avoid them. We suggest that these concerns about antibiotics form a resource for educating patients, for health promotion and social marketing strategies.

## 1. Introduction

Antibiotic resistance is a serious and growing threat to public health in both developing and developed countries [1,2]. Much of this is due to inappropriate prescribing and use of antibiotics for conditions for which they are ineffective (such as viral upper respiratory tract infections) [3]. Lay people’s understanding and knowledge of antibiotics are important because they are a significant determinant of inappropriate use. Patients either access antibiotics directly without a prescription (although this is illegal in most countries, it is common practice, and not solely in developing countries [2,4,5,6]) or they can request antibiotics from physicians. Patient demand, or at least doctors’ perception of patient demand [7], leads to increased prescribing [8,9,10,11].

Numerous studies have found limited public knowledge and understanding of antibiotics and limited understanding of conditions for which they are useful or not useful [12,13,14,15,16,17]. Few members of the public appear to have a good understanding of, or are particularly concerned about antibiotic resistance [15,18,19]. Studies looking at public knowledge and understanding of resistant organisms such as Methicillin Resistant *Staphlycoccus aureus* (MRSA) suggest that the public do not usually perceive any link between “superbugs” like MRSA and antibiotic prescribing or use [20,21,22].

Many interventions have been developed to attempt to reduce inappropriate prescribing and use. Those that have been successful have had limited impact, and may not be sustainable in the longer term [23,24]. It has been suggested that almost all previous initiatives have been grounded in a traditional, information-intensive health education approach that relies on changing knowledge, and assumes that this changed knowledge will automatically lead to changes in behavior [24]. The assumption that knowledge leads to behavior change is problematic, and even if it does, behavior change is likely to be short-term “unless motivators and values become firmly rooted and norms that support lasting change are established within populations” [24]. Health promotion approaches stress the need to listen to and involve target groups in behavior change [25], and both health promotion and social marketing approaches stress the need to understand and listen to people’s current views and ways of talking about an issue, before designing campaigns to change their behavior. In order to develop effective patient education and health promotion to reduce inappropriate antibiotic use we need to understand how people talk about and think about antibiotics and infection [24,25]. Previous studies have focused on what motivates inappropriate demand for and use of antibiotics, and have tended to ignore existing views and practices that could form resources for reducing inappropriate use [12,13,14,15,16,17]. These include perceptions of the negative consequences of antibiotic use, lay people’s reluctance to use antibiotics, and the strategies they use to avoid antibiotics. These could form a useful basis for developing educational and health promotion interventions that are relevant and acceptable to the target population and could result in new culturally rooted norms about antibiotics and their use.

Existing educational campaigns about antibiotics frequently stress the difference between bacterial and viral illnesses, but research suggests that most members of the public do not understand this distinction [17,26]. Media and public education campaigns about antibiotics often use military metaphors. For example, using the analogy of infection as war, pathogenic bacteria as invaders, the body fighting infection, using illustrations that portray antibiotics as soldiers in a war, terms like an arms race against bacteria, but there is little evidence about whether such metaphors feature in the understandings of members of the public [27].

The aim of this study is to explore how European New Zealanders think about and talk about antibiotics, and their understandings of infection and resistance that are likely to affect antibiotic use.

## 2. Methods

This study is part of a wider project on Medications in Everyday Life, which used qualitative methods to explore how people think about and use medicines [28,29,30]. Qualitative methods were chosen to allow the researchers to explore the meanings of medications and their use in households in depth, and to respond to participants’ views of what is important without imposing pre-conceived ideas about the topics. Ethnographic methods were used to collect data from 7 focus groups, and in addition, 59 households. Households rather than individuals were the primary unit of data-gathering so that we could explore relationships between household members around medicine-taking and explore the storage and use of medicines within households. Previous research on medicine-taking has focused on individuals and may consequently have neglected the effects of relationships on medicines use, and the effect of medicines use on relationships. We specifically sought different types of households: those with children, those where one or more member has a chronic illness, those who used complementary and alternative medicines, and households of a specific ethnicity (e.g., Tongan households). Participant households which met these criteria were purposefully sampled. This is appropriate in qualitative research, where the aim is not to obtain a statistically representative sample and make statistical inferences from the results, but rather to obtain an information rich sample and make logical inferences from that sample. Households were recruited through informal networks, advertising, and through health-related organizations. For example, to recruit those with a chronic illness, organizations representing people with illnesses were asked to suggest potential participants. Research team members from particular ethnic groups recruited households through their informal networks and through snowball sampling. However, many of the participant households fitted into more than one category, for example, they may have children, use alternative medicines and have a member with a chronic illness. A range of types of households were included, such as couples, single parents with children, couples with children, and three generation households. Participants were from a range of socio-economic positions such as professional and non-professional occupations, retired people, people reliant on unemployment or other benefits. Within each participating household, all household members participated in the research. Most participating households volunteered by responding to an advertisement or request from an agency or a friend or colleague. Thus only a very small number of households refused to participate after finding out more about the demands of the project. A NZ$100 grocery voucher was given to households who participated, in recognition of the substantial time commitment required of them. Focus group participants were given $10 each to compensate for travel costs. Demographic details of the households and focus groups are described in Table 1.

**Table 1:** Characteristics of participants.

Focus groups (n = 4 groups, n = 21 participants)	
Age	Mean: 47.7
Gender	Male: 8, Female: 13
Household members (n = 39 households, n = 101 participants)	
Age	Mean: 34, SD: 21.8
Gender	Male: 60, Female: 41
Household type	Couple: 8 Couple with child or children: 12 Single parent with child or children: 7 Person living along: 7 Other: 5

A wide range of methods such as household interviews, photo-elicitation interviews (where participants were given a camera, asked to take medicine-related photos, and to discuss these with the interviewer), were included. Two types of diaries were kept: diaries of medicine taking and diaries of any contact with medication (such as advertising). In both cases these were followed by interviews to discuss what participants had recorded. This paper draws on focus groups, and individual or group interviews with households. Although the study included people from a range of ethnicities, only the four focus groups and in addition, thirty-nine households with members of New Zealand European descent were included in this paper, because it is likely that ethnicity affects how people think about and talk about medicines [14,31].

Transcripts of focus groups and interviews were imported into NVivo [32]. Since the focus of the analysis was on how people talked about key concepts, the first part of the analysis involved finding these concepts in the data. Key words were identified a priori: “infection”, “antibiotic”, “penicillin”, “bacteria”, “virus”, “immune”, “immunity”, and “resistant”, “resistance”, “bug”. Text which included one (or more) of those key words was identified using NVivo’s Text Search Query function, and choosing the broad context option. The results were automatically coded under tree nodes with these names (with very similar words like immune and immunity coded in one node). The second part of the analysis involved manual coding in NVivo, to identify themes/ways of using or talking about each of these concepts. All material was read at least twice by the first author to improve consistency of coding. Where excerpts were not clear because too little material had been extracted by the text search function, the original transcript was referred back to. During analysis the text search function was used to look at additional words which could answer questions raised by the analysis.

Ethical approval for this study was granted by Massey University Human Ethics Committee (MUHECN 08/067, dated 23 October 2008). Participants gave informed consent before participating in the study. To protect participant anonymity, pseudonyms are used throughout this paper.

## 3. Results and Discussion

Although the study was about medication in general (including non-prescription, alternative medicines and supplements) antibiotics were frequently mentioned. Although some people spoke of antibiotics, particularly topical formulations, as unproblematic routine items, it was clear that many people in the study had a strong inclination to avoid oral antibiotics. The initial section of results therefore considers how people express aversion to antibiotic use, and then considers strategies that were used to avoid antibiotic use while preventing and managing infection. Following that, we discuss a range of related matters discussed by participants that are relevant to antibiotic use, namely, metaphors of infection and conceptions of immunity and resistance.

### 3.1. Aversion to Taking Antibiotics

For many people in the study, their aversion to taking antibiotics reflected a general reluctance to take medicines [29]. Their goal if they had an infection was to manage it without taking antibiotics. Amongst these people, even those who knew they needed an antibiotic took it with resignation rather than enthusiasm. For example, Janice had a heart valve problem that meant that she needed prophylactic antibiotics before dentistry. She said: “part of me thinks, oh, I don’t want to do this.” Meg’s son needed antibiotics when he had an operation for severe appendicitis, and she said: “sometimes you just have to”.

The frequent use of antibiotics for recurrent infections in children was particularly troubling for many parents. Susan said that repeated use of antibiotics is “just so not good for you.” Experiences of adverse effects also made parents reluctant to use antibiotics. Paula’s baby son had suffered from vomiting and diarrhea last time he took antibiotics, so she was quite reluctant to give him another course later. Jemma said “they had upset tummies with them and they didn’t like the taste of them so it was a real battle to get it into them and I just felt more comfortable using homeopathy. Particularly when they got sick often.” 

Several participants had experienced allergic reactions to antibiotics in the past. One had had a life-threatening reaction when she was a teenager. These and other adverse effects, such as fungal infections, contributed to reluctance to take antibiotics. One of the adverse effects commonly attributed to antibiotics was that they upset the body’s balance somehow. For most people this was the balance of good and bad bacteria in the gastro-intestinal tract. Some participants mentioned that antibiotics kill both good and bad bacteria and that something needs to be done to restore the balance of these. Strategies included changing diet and taking probiotics. These were thought to reduce gastro-intestinal problems and thrush that may result from taking antibiotics, and to generally restore the body’s balance. Trish was less specific but knew of a product to “keep your insides healthy” after taking antibiotics. Meg talked more generally about “correcting that damage” caused by taking an antibiotic.

Some people were staunchly opposed to antibiotics and refused to take them. Mary said “They hand out antibiotics for everything, just even to have teeth out—antibiotics. You don’t need them.” However most were resigned to taking them if they thought the infection was serious, or if other strategies (see below) were not working. Warren said that he did not like taking antibiotics, and he believed that many other people took them unnecessarily. “I can name six guys out of 15 that I work with that regularly with coughs and colds and that are always down to the doctor to get antibiotics.” He said: “I never do that. I don’t think things like that need antibiotics. I think antibiotics should be saved for dire emergencies”.

Andrew thought that over-use of antibiotics has led to children experiencing much more illness than when he was a child. Brett thought that the disinfectant products advertised on television are fuelling paranoia about bacteria, when bacteria are necessary for our survival.

### 3.2. Strategies for Preventing and Dealing with Infection

In the interviews, people described a range of strategies for dealing with infection that did not involve antibiotics. Those who had experienced (or whose family members had experienced) recurrent infections had developed strategies that they believed prevented these. Many, but by no means all of these households, were interested in, or used alternative medicines.

A wide range of strategies were described for treating colds and flu, and preventing these becoming more problematic. These included rest, using paracetamol, lozenges for sore throats available without prescription, cough mixture, gargling grapefruit seed extract or tea tree oil for sore throats, eating garlic at first symptom of possible cold, homemade cough syrup, and blends of essential oils developed especially to deal with cold and flu symptoms, such as a ”chest rub” blend. Ear infections were very concerning for some people, particularly if they were recurrent. Participants reported using homeopathic remedies and (naturopathic) earache relief drops. Those who experienced recurrent ear and sinus infections (either themselves or their children) had developed strategies for preventing these. These included xylitol nasal spray, or syrup, nasal sprays (including saline or plant based sprays) and other devices for nasal irrigation, and taking an antihistamine to prevent post-nasal drip. These aimed to clear sinuses and airways to prevent infection developing.

For urinary tract infections, participants reported taking grapefruit seed extract orally, urinary alkilinizer sachets, drinking cranberry juice, and taking homeopathic drops, all to prevent recurrent infections. For stomach “bugs” people using oral rehydration solution, eating charcoal, and being fastidious with hand washing to prevent the spread of the infection. For skin infections or wounds, strategies included putting crushed garlic straight onto a wound, using lavender oil as an antiseptic for bites, making a sugar and soap poultice, taking silica tablets for boils, and allowing a dog to lick wounds. Sources of these strategies varied, but were often family, friends or material participants had read.

### 3.3. Ways of Talking about Infections, Antibiotics, and Resistance

Although military metaphors are frequently used in health promotion, there was very little if any use of military metaphors by our participants. Sheila talked about “getting the body’s immune system to be strong enough to defend whatever attacks it”, but this was uncommon. There was some limited use of fighting metaphors, but these were not military, involving armies of invading microbes engaged in battles with the body’s own armies. Rather, they seemed to reflect a much more personal hand-to-hand combat. For example, Carole said: “I’ve been trying to fight off this bug”. Hitting/knocking/belting an infection on the head were also used. To justify her decision to get antibiotics for her child, Paula said “Why wait more days … When you can knock it on the head?” Jemma also talked of knocking an infection on the head. Ben said: “I kind of see antibiotics as the punch” and also said that antibiotics will “belt an infection over the head.” Brendan reported using antibiotics “to give it [an infection] a kick”. A focus group participant explained why it was important to finish a whole course of antibiotics: “otherwise it will mutate and it will come back and bite you in the ass next time”. Geraldine spoke of antibiotics killing bacteria, but she said they kill both good and bad bacteria. Fleur said charcoal fixes stomach bugs because “It sucks up the beasties.” 

In contrast to military metaphors, which were rarely used, the idea of *clearing* an infection was commonly used by participants. For example, Paula said her son has taken two courses of antibiotics for his chest infection, but “it still hasn’t cleared up”. Rosie said she took antibiotics for her chest infection and they “cleared it up”. She used “clearing up” as a way of describing effective treatment of an infection throughout her interview. Natasha said her friend was given a “back pocket” prescription for antibiotics to use “if the attack didn’t clear”. Warren believed that taking an antibiotic “doesn’t clear it up any quicker”. The notion of “clearing” here seems to be used in the sense of removing something inappropriate or bad from a space where it is not supposed to be [33]. In this case the bad/inappropriate things are infection or products produced because of an infection (primarily mucus). 

Participants used the terms “immunity” and “resistance” very frequently, but these were used in different senses from those in medical/scientific language. They also used these terms in different ways when talking about resistance to medication than when talking about resistance to disease. We discuss these in turn.

In talking about medications, there was one sense in which participants appeared to use the words “immunity” and “resistance” interchangeably. This was to refer to the concept that the body becomes accustomed to medication, affecting the body’s normal function in some way. Usually this meant that medicines became less effective. They applied both to antibiotics and other medications. For example, Jasmine takes many drugs for on-going mental health problems. When she has stopped these medicines in the past she has become extremely unwell, and she attributed this to her body being “immune” to the medicines, that is, so accustomed to them that it is unable to function properly without them. Julie said her friend takes a lot of paracetamol but claims that it does not work, which Julie attributed to her becoming immune to the paracetamol. Jack said that paracetamol ceased to be effective for his headaches because he developed a resistance to it, so he “weaned” himself off it, and now a lower dose is effective again.

The possibility of the body becoming immune or resistant to antibiotics deterred some people from taking antibiotics. May was not sure of the terminology, but tried to avoid antibiotics because she is “a bit worried about being immune to it or whatever it’s called”. Tina had tonsillitis as a child and said “years and years and years of tonsillitis and I became immune to the antibiotics. They did nothing”. “Similarly Warren believed that “I know doctors keep saying that the antibiotics keep changing and as long as you take the course your body’s not getting immune to them, but I think the less you take them the better off you are.” Rosie believed that antibiotics were no longer working for her friend, because “after a while your body just gets used to it”.

It appears that these people were using the idea of being immune or resistant to antibiotics in the same way as they used those terms for other drugs (that the body became accustomed to them so they became less effective). For them, immunity or resistance is a property of humans, rather than of bacteria. Very few people mentioned resistance as a property of microbes. This did not appear to be a commonly understood concept. One very infrequent exception to this was Mel, who said “if you don’t take the whole dose of antibiotic yourself, you know, there’s no effect … They say it strengthens the germ and so next time you get it you might get rid of it then it’ll be twice the resistance.”

Much of the talk focussed more generally on immunity and resistance to disease. Participants frequently mentioned the immune system or “resistance”, the ability of humans to resist infections and disease. This seemed to be different from the use of “resistance” (described above) to refer to medicines becoming less effective as the body becomes accustomed to them. The main context in which participants discussed the immune system was to talk about the need to strengthen it. Strengthening the immune system tended to be regarded positively, compared to treating infections, which participants tended to regard as a less favorable option. Strengthening the immune system was regarded as a more holistic approach, compared with the “quick fix” of treating an infection.

Strengthening the immune system was sometimes described as “supporting” it: Janice said: “The multi-vitamins support, they don’t replace what you’re not getting through your food but I see them that they just support you for whatever reason that you’re taking them. Whereas the antibiotics are to cure something”. Verity said: “what you’re doing is supporting your body’s own natural defences”. Our participants stressed the importance of rest, sleep and good food, but also mentioned a wide range of products to strengthen the immune system. These included multivitamins, cod liver oil, vitamin D, olive leaf extract, echinacea, traditional Ayurvedic remedies, malt extract, specific products with names like “Child Immune Care”, or “Flora Immune” and essential oils (for massage or burning). Allowing children to experience illness and get better naturally was also considered to be good for the immune system by some participants. Naturopaths and homeopaths were consulted by some participants either to provide products to reduce susceptibility to infection or to build themselves up after an infection.

There was only one instance in which an immune system was conceptualized as being too strong. A woman with haemolytic anaemic said that her immune system was killing off her red blood cells. Allergies or autoimmune diseases were not conceptualized as an immune system that was over-reactive or reacting to the wrong things.

## 4. Conclusions

Previous research on lay people’s understanding and use of antibiotics has focused on patient demand for antibiotics, and tended to ignore people’s concerns about taking antibiotics and the strategies they use to avoid taking them [12,13,14,15,16,17]. Participants in this study were worried about adverse effects of antibiotics, particularly for recurrent infections. Some were concerned that antibiotics upset the body’s balance in some way, and many used a range of strategies to try to prevent and treat infections without antibiotics. They tended to believe that it was better to strengthen the body to resist infection than to treat it. They tended not to use military metaphors but instead spoke of clearing infections, and had little understanding of antibiotic resistance but thought that over-using antibiotics was unwise because it would reduce their effectiveness in future.

Concerns about taking prescription medicines have been documented in many qualitative studies [34], although this is infrequently highlighted in studies of antibiotic use. Pechère also found that most people (59%) in an international study believed that antibiotics undermined immunity. A quarter of participants had had side effects from taking antibiotics, and some also described antibiotics as unpredictable, mysterious, aggressive, and/or frightening [6]. Hawkings *et al*. [35] found around a quarter of their respondents tried to avoid antibiotics, in some cases because they thought that their bodies might become used to them, making them ineffective. We found such beliefs to be widespread in our study, which took place in a different cultural context, and was able to explore these views in more detail than previous studies. However, Haynes *et al*. [16] found that most people believed that antibiotics were effective for common colds, particularly when discoloured nasal discharge was present, similar numbers also reported antihistamines, decongestants, and pain relievers to be effective. Chicken soup, breathing steam and Vitamin C were reported to be effective by fewer people, but were still believed to be effective by 35% to 50% of participants. It is well established that most symptoms experienced on a day-to-day basis are managed initially within the home without formal healthcare [36,37,38]. This study shows that people use these kinds of strategies to avoid antibiotic use. 

Others have also noted that lay people often think of antibiotic resistance as a characteristic of people rather than bacteria: this has important implications for designing interventions, because if resistance is a personal characteristic excessive antibiotic use puts only the individual at risk, not others [20,35,39,40].

There is a long history in New Zealand of emphasis on building up the body to resist illness. Bagge discussed the extensive use of tonics (particularly products containing malt or cod liver oil) around the middle of the twentieth century in New Zealand to “build people up” or strength them [41]. This view of health, which stresses susceptibility to infection, rather than the presence or activity of an infectious agent, was described by Payer [42] as being characteristic of the French, rather than English approaches to health. In our study people did not appear to believe immune system could be too strong or over-reactive. Allergies were not spoken of in this way, as they have been in other studies [27].

Limitations of the study include the non-random sample. The study demanded a high level of participation from households and it is likely that people who were more interested in the topic of medicines were more likely to volunteer. Participants represented a range of socio-economic positions and ages. It is also possible that people with alternative views about health and illness are over-represented in the study, but it is important to note that much of the material in the paper is drawn from households that were not recruited because of their use of alternative medicines. Coding was done by only one author, and relied heavily on the text search feature of NVivo.

Studies which examine public views of antibiotics tend to focus on problems such as lack of knowledge, or belief in the curative powers of antibiotics for viral illness, and neglect the other side of the coin, that is, the concerns that people have about antibiotics, and the fact that many people try to avoid them. We suggest that these views form a resource for patient education and health promotion.

Interventions to reduce the inappropriate use of antibiotics need to be based in sound knowledge of what people already know and do about infection. This study shows that considerable reluctance to take antibiotics already exists. This could be used as the basis for interventions. For example, the Australian campaign, “Common colds need common sense” stressed rest and symptom management, building on existing community responses to colds [43] This is also consistent with WHO’s recommendation that traditional medicines (in this case home remedies) should be recognized as a resource, and that member states should consider including traditional medicine into their national health systems, based on evidence of safety, efficacy and quality [44]. Campaigns which stress and build on traditional ways of avoiding infection, building up resistance and treating minor infections may be more successful than those which attempt directly to limit antibiotic use.

## References

[1] American Academy of Microbiology (2009). Antibiotic Resistance: An Ecological Perspective on an Old Problem.

[2] Okeke I.N., Laxminarayan R., Bhutta Z.A., Duse A.G., Jenkins P., O’Brien T.F., Pablos-Mendez A., Klugman K.P. (2005). Antimicrobial resistance in developing countries. Part I: Recent trends and current status. Lancet Infect. Dis..

[3] Standing Medical Advisory Committee Sub-Group on Antimicrobial Resistance (1998). The Path of Least Resistance.

[4] Dameh M., Norris P., Green J. (2010). Antibiotic sales at private pharmacies in Abu Dhabi, United Arab Emirates. Pharm. World Sci..

[5] Väänänen M.H., Pietilä K., Airaksinen M. (2006). Self-medication with antibiotics—Does it really happen in Europe?. Health Policy.

[6] Pechère J. (2001). Patients’ interviews and misuse of antibiotics. Clin. Infect. Dis..

[7] Britten N. (1994). Patient demand for prescriptions: A view from the other side. Fam. Pract..

[8] Miller E., MacKeigan L., Rosser W., Marshman J. (1999). Effects of perceived patient demand on prescribing anti-infective drugs. CMAJ.

[9] Coenen S., Michiels B., Renard D., Denekens J., van Royen P. (2006). Antibiotic prescribing for acute cough: The effect of perceived patient demand. Br. J. Gen. Pract..

[10] Stevenson F.A., Greenfield S.M., Jones M., Nayak A., Bradley C.P. (1999). GPs’ perceptions of patient influence on prescribing. Fam. Pract..

[11] Britten N., Ukoumunne O. (1997). The influence of patients’ hopes of receiving a prescription on doctors’ perceptions and the decision to prescribe: A questionnaire survey. Br. Med. J..

[12] Norris P., Chong C., Chou A., Hsu T., Lee C., Su C., Wang Y. (2009). Knowledge and reported use of antibiotics amongst school-teachers in New Zealand. Pharm. Pract..

[13] Norris P., Churchward M., Fa’alau F., Va’ai C., Arroll B. (2009). Understanding and use of antibiotics amongst Samoan people in New Zealand. J. Prim. Health Care.

[14] Norris P., Ng L., Kershaw V., Hanna F., Wong A., Talekar M., Oh J., Azer M., Cheong L. (2009). Knowledge and reported use of antibiotics amongst immigrant ethnic groups in New Zealand. J. Immigr. Minor. Health.

[15] Eng J.V., Marcus R., Hadler J.L., Imhoff B., Vugia D.J., Cieslak P.R., Zell E., Deneen V., McCombs K.G., Zansky S.M. (2003). Consumer attitudes and use of antibiotics. Emerg. Infect. Dis..

[16] Haynes D., Mainous A., Oler M., Zoorob R. (1997). Patient knowledge of upper respiratory infections: Implications for antibiotic expectations and unnecessary utilization. J. Fam. Pract..

[17] Wilson A.A., Crane L.A., Barrett P.H., Gonzales R. (1999). Public beliefs and use of antibiotics for acute respiratory illness. J. Gen. Intern. Med..

[18] Hawkings N., Wood F., Butler C. (2007). Public attitudes towards bacterial resistance: A qualitative study. J. Antimicrob. Chemother..

[19] Rodis J.L., Green C.G., Cook S.C., Pedersen C.A. (2004). Effects of a pharmacist-initiated educational intervention on patient knowledge about the appropriate use of antibiotics. Am. J. Health-Syst. Pharm..

[20] Brooks L., Shaw A., Sharp D., Hay A. (2008). Towards a better understanding of patients’ perspectives of antibiotic resistance and MRSA: A qualitative study. Fam. Pract..

[21] Gould D.J., Drey N.S., Millar M., Wilks M., Chamney M. (2009). Patients and the public: Knowledge, sources of information and perceptions about healthcare-associated infection. J. Hosp. Infect..

[22] Easton P.M., Marwick C.A., Williams F.L.R., Stringer K., McCowan C., Davey P., Nathwani D. (2009). A survey on public knowledge and perceptions of methicillin-resistant staphylococcus aureus. J. Antimicrob. Chemother..

[23] Arnold S., Straus S. (2005). Interventions to improve antibiotic prescribing practices in ambulatory care. Cochrane Database Syst. Rev..

[24] Edgar T., Boyd S., Palame M. (2009). Sustainability for behaviour change in the fight against antibiotic resistance: A social marketing framework. J. Antimicrob. Chemother..

[25] Fresle D., Wolfheim C. (1997). Public Education in Rational Drug Use: A Global Survey.

[26] Arroll B., Everts N. (1999). The common cold: What does the public think and want?. N Z Fam. Physician.

[27] Martin E. (1994). Flexible Bodies: Tracking Immunity in American Culture: From the Days of Polio to the Age of Aids.

[28] Dew K., Chamberlain K., Hodgetts D., Norris P., Radley A., Gabe J. (2013). Home as a hybrid centre of medication practice. Sociol. Health Illn..

[29] Chamberlain K., Madden H., Gabe J., Dew K., Norris P. (2011). Forms of resistance to medications within New Zealand households. Medische Antropologie.

[30] Hodgetts D., Chamberlain K., Gabe J., Dew K., Radley A., Madden H., Norris P., Nikora L. (2011). Emplacement and everyday use of medications in domestic dwellings. Health Place.

[31] Nichter M., Nichter M. (1996). Anthropology and International Health: Asian Case Studies.

[32] NVivo (2008).

[33] Oxford English Dictionary. http://www.oed.com/.

[34] Pound P., Britten N., Morgan M., Yardley L., Pope C., Daker-White G., Campbell R. (2005). Resisting medicines: A synthesis of qualitative studies of medicine taking. Soc. Sci. Med..

[35] Hawkings N., Butler C., Wood F. (2008). Antibiotics in the community: A typology of user behaviours. Patient Educ. Couns..

[36] Verbrugge L., Ascione F. (1987). Exploring the iceberg: Common symptoms and how people care for them. Med. Care.

[37] Cunningham-Burley S., Irvine S. (1987). “And have you done anything so far?” An examination of lay treatment of children’s symptoms. Br. Med. J..

[38] Rogers A., Hassell K., Nicolaas G. (1999). Demanding Patients: Analysing the Use of Primary Care.

[39] Brookes-Howell L., Elwyn G., Hood K., Wood F., Cooper L., Goossens H., Ieven M., Butler C. (2011). “The body gets used to them” patients’ interpretations of antibiotic resistance and the implications for containment strategies. J. Gen. Intern. Med..

[40] Norris P., Toner E., Morris K. (2003). Public understandings of bacteria, antibiotics and resistance. N Z Med. J..

[41] Bagge M. (2012). Medicines in the Context of Older People’s Lives.

[42] Payer L. (1988). Medicine and Culture: Varieties of Treatment in the United States, England, West Germany, and France.

[43] National Prescribing Service (NPS) Australia MedicineWise Common Cold. http://www.nps.org.au/bemedicinewise/common_colds.

[44] Executive Board WHO (2009). Traditional Medicine.

